# Depurinating estrogen-DNA adducts, generators of cancer initiation: their minimization leads to cancer prevention

**DOI:** 10.1186/s40169-016-0088-3

**Published:** 2016-03-15

**Authors:** Ercole L. Cavalieri, Eleanor G. Rogan

**Affiliations:** Eppley Institute for Research in Cancer and Allied Diseases, University of Nebraska Medical Center, Omaha, NE USA; Department of Environmental, Agricultural and Occupational Health, College of Public Health, University of Nebraska Medical Center, Omaha, NE USA

**Keywords:** Estrogen metabolism, Catechol estrogen-3,4-quinones, Depurinating estrogen-DNA adducts, Estrogen genotoxicity, Estrogen carcinogenesis, Cancer prevention, *N*-acetylcysteine, Resveratrol

## Abstract

Estrogens can initiate cancer by reacting with DNA. Specific metabolites of endogenous estrogens, the catechol estrogen-3,4-quinones, react with DNA to form depurinating estrogen-DNA adducts. Loss of these adducts leaves apurinic sites in the DNA, generating mutations that can lead to the initiation of cancer. A variety of endogenous and exogenous factors can disrupt estrogen homeostasis, which is the normal balance between estrogen activating and protective enzymes. In fact, if estrogen metabolism becomes unbalanced and generates excessive catechol estrogen 3,4-quinones, formation of depurinating estrogen-DNA adducts increases and the risk of initiating cancer is greater. The levels of depurinating estrogen-DNA adducts are high in women diagnosed with breast cancer and those at high risk for the disease. High levels of depurinating estrogen-DNA adducts before the presence of breast cancer indicates that adduct formation is a critical factor in breast cancer initiation. Women with thyroid or ovarian cancer also have high levels of estrogen-DNA adducts, as do men with prostate cancer or non-Hodgkin lymphoma. Depurinating estrogen-DNA adducts are initiators of many prevalent types of human cancer. These findings and other discoveries led to the recognition that reducing the levels of estrogen-DNA adducts could prevent the initiation of human cancer. The dietary supplements *N*-acetylcysteine and resveratrol inhibit formation of estrogen-DNA adducts in cultured human breast cells and in women. These results suggest that the two supplements offer an approach to reducing the risk of developing various prevalent types of human cancer.

**Graphical abstract:**
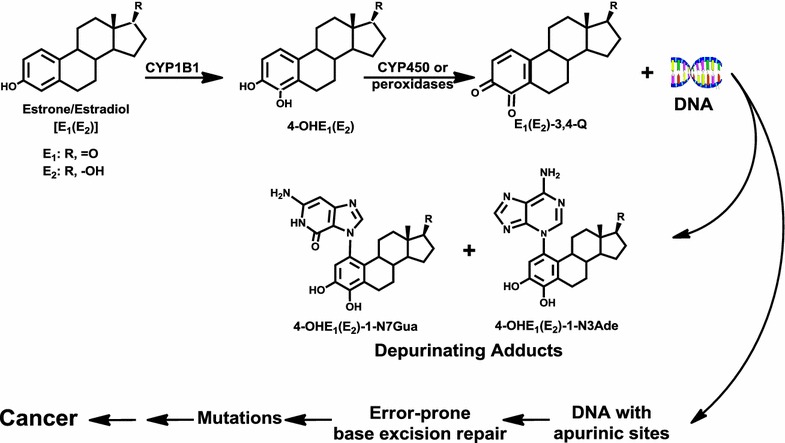
Major metabolic pathway in cancer initiation by estrogens.

Major metabolic pathway in cancer initiation by estrogens.

## Mechanism of metabolic activation of estrogens to initiate cancer

One of the major obstacles in cancer research is related to the concept that cancer is a problem of many diseases. This viewpoint has kept researchers from investigating the etiology of cancer because a search for numerous causes would be prohibitively complex. While the expression of various cancers coincides with the concept of numerous diseases, we think many types of prevalent cancers have a common etiology. The understanding of this common mechanism of cancer initiation can lead to cancer prevention.

Exposure to estrogens is a known risk factor for developing cancer. The scientific community predominantly considers estrogens to be epigenetic carcinogens because these compounds do not induce mutations in standard bacterial and mammalian test systems. This presumably occurs because the reactive catechol estrogen quinone metabolites are not formed or cannot reach the target DNA [1–5]. These results have led scientists to classify estrone (E_1_) and estradiol (E_2_) as epigenetic carcinogens that function by stimulating abnormal cell proliferation via estrogen receptor (ER)-mediated processes [5–10]. These latter events can accelerate the process of carcinogenesis, but do not play the critical role in cancer initiation because the hypothetical mutations obtained are random.

Unbalanced estrogen metabolism is a critical factor in cancer initiation. The discovery that specific oxidative metabolites of estrogens, the catechol estrogen quinones, react with DNA supports the hypothesis that estrogens can become endogenous carcinogens by generating the mutations leading to the initiation of cancer [11–14]. This paradigm indicates that specific, critical mutations produce abnormal cell proliferation leading to cancer, rather than ER-mediated abnormal cell proliferation that generates random mutations [1, 6–10]. The specificity of the critical mutations is the result of the preliminary intercalating physical complex between estrogen and DNA that occurs before formation of the covalent bond between them. This intercalating mechanism has been demonstrated for the human carcinogen diethylstilbestrol (DES) [15].

### Benzene and naphthalene

**Fig. 1:**
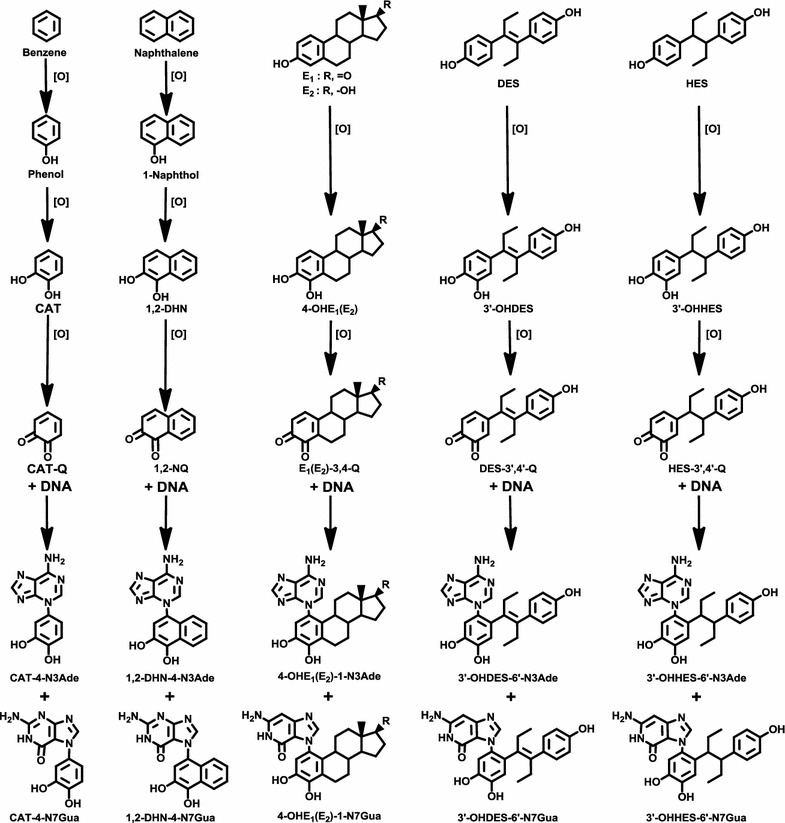
Common mechanism of metabolic activation and reaction with DNA to form the N3Ade and N7Gua depurinating adducts for benzene, naphthalene, estrone (E_1_), estradiol (E_2_), diethylstilbestrol (DES), and hexestrol (HES). The figure shows the progression from parent compound to hydroxy derivative, catechol, and then quinone, which reacts with DNA to form the depurinating adducts at the N3Ade and N7Gua

Natural and synthetic estrogens contain a benzene ring in their structure. For compounds containing one or two benzene rings, there is a common mechanism of metabolic activation, which produces extremely weak ultimate carcinogens. This mechanism of activation (Fig. 1) has been demonstrated to occur with benzene [16, 17], naphthalene [18, 19], the natural estrogens E_1_ and E_2_ [20–26], and the synthetic estrogens DES [15, 27] and hexestrol (HES) [23, 28, 29].

It has long been known that benzene causes acute myelogenous leukemia in humans [30, 31]. The benzene metabolites include catechol, (CAT, 1,2-dihydroxybenzene) and hydroquinone (1,4-dihydroxybenzene) [32, 33]. CAT and hydroquinone can accumulate in the bone marrow [34, 35], where they can be oxidized by peroxidases [36] to the corresponding quinones. The CAT-1,2 quinone reacts with DNA by 1,4 Michael addition to yield the depurinating adducts CAT-4-N7Gua and CAT-4-N3Ade (Fig. 1) [16, 17]. These results suggest that the ultimate carcinogenic metabolite of benzene is the benzene-1,2-quinone.

Inhalation of naphthalene by male and female rats for two years produced olfactory epithelial neuroblastomas in 5–10 % of the animals [37]. The logical mechanism of metabolic activation of naphthalene is analogous to the one described above for benzene. In fact, naphthalene-1,2-quinone reacts with DNA to produce the depurinating N3Ade and N7Gua adducts in vitro and in vivo (Fig. 1) [18, 19].

### Natural and synthetic estrogens

One of the major metabolic pathways of the natural estrogens E_1_ and E_2_ is the formation of catechol estrogens. These metabolites are oxidized to semiquinones and then to quinones. Their reaction with DNA forms predominantly the depurinating adducts N3Ade and N7Gua that can initiate cancer (Fig. 1). Synthetic estrogens, such as the human carcinogen DES [38] and its hydrogenated derivative HES, display properties similar to the natural estrogens: (1) they are carcinogenic in the kidney of Syrian golden hamsters [39, 40]; (2) the major metabolites are their catechols [40–43]; (3) the catechol quinones of DES and HES have chemical and biochemical properties similar to those of the natural E_1_(E_2_)-3,4-quinones [E_1_(E_2_)-3,4-Q], namely they form N3Ade and N7Gua adducts after reaction with DNA (Fig. 1). Depurination of the N3Ade adduct is instantaneous, whereas depurination of the N7Gua adduct occurs rather slowly [15, 23, 27–29]. These data suggest that the catechol quinones of DES and HES are their cancer initiators.

## Catechol estrogen metabolic pathway

Strong evidence from studies of estrogen metabolism, formation of DNA adducts, mutagenicity, cell transformation and carcinogenicity led to and supports the hypothesis that specific estrogen metabolites, the catechol estrogen quinones, can react with DNA to form estrogen-DNA adducts in critical genes that lead to the initiation of cancer [11, 12].

**Fig. 2:**
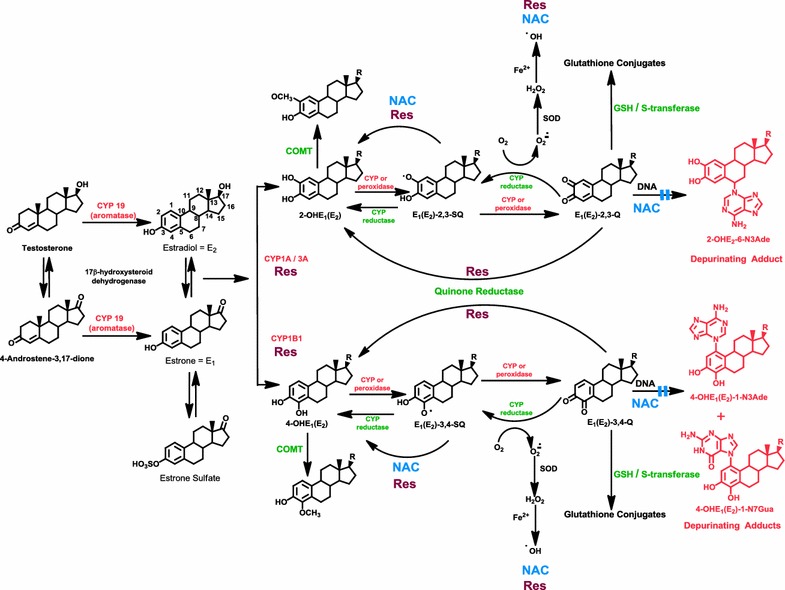
Formation of estrogens, catechol estrogen metabolic pathway and DNA adducts of estrogens. Activating enzymes and depurinating DNA adducts are in red and protective enzymes are in *green*. *N*-acetylcysteine (NAC, shown in *blue*) and resveratrol (Res, shown in *burgundy*) indicate the various steps where NAC and Res can improve unbalanced estrogen metabolism by reducing formation of depurinating estrogen-DNA adducts

Metabolic formation of estrogens derives from aromatization of testosterone and 4-androstene-3,17-dione, catalyzed by CYP19 (aromatase), to yield E_2_ and E_1_, respectively (Fig. 2). E_2_ and E_1_ are interconverted by 17β-hydroxysteroid dehydrogenase. If an excess of estrogen is obtained, it is stored as estrone sulfate. Estrogens are metabolized via two major pathways: formation of 16α-OHE_1_(E_2_) (not shown in Fig. 2) and formation of the catechol estrogens 2-OHE_1_(E_2_) and 4-OHE_1_(E_2_) (Fig. 2) [44]. Cytochrome P450 (CYP)1A and CYP3A catalyze the hydroxylation preferentially at the 2 position, whereas CYP1B1 catalyzes the hydroxylation almost exclusively at the 4 position [45–47]. The two catechol estrogens are inactivated by conjugation to glucuronides and sulfates especially in the liver (not shown in Fig. 2). In extrahepatic tissues, the most common path of conjugation of the catechol estrogens is *O*-methylation, catalyzed by catechol-*O*-methyltransferase (COMT) [48, 49]. A low activity of COMT renders more competitive oxidation of the catechol estrogens to E_1_(E_2_)-2,3-Q and E_1_(E_2_)-3,4-Q catalyzed by CYP or peroxidases (Fig. 2).

Oxidation of semiquinones to quinones can also be obtained by molecular oxygen (Fig. 2). Reduction of estrogen quinones to semiquinones by CYP reductase completes the redox cycle. In this process, the molecular oxygen is reduced to superoxide anion radical, and then converted by superoxide dismutase to hydrogen peroxide. In the presence of Fe^2+^ the hydrogen peroxide is converted to hydroxyl radical. Reaction of the hydroxyl radical with lipids produces lipid hydroperoxides [50] (not shown in Fig. 2).

Following the formation of catechol estrogen quinones (Fig. 2), they can be inactivated by reacting with glutathione (GSH). A further inactivation pathway for the quinones is reduction to their respective catechols by quinone reductase [51, 52], a protective enzyme that can be induced by a variety of compounds [53].

If all the protective processes are insufficient, the catechol estrogen quinones can react with DNA to form predominantly the depurinating adducts 4-OHE_1_(E_2_)-1-N3Ade plus 4-OHE_1_(E_2_)-1-N7Gua (97 %) from E_1_(E_2_)-3,4-Q and 2-OHE_1_(E_2_)-6-N3Ade (3 %) from E_1_(E_2_)-2,3-Q (Fig. 2). The much larger amount of adducts formed by the E_1_(E_2_)-3,4-Q compared to those from the E_1_(E_2_)-2,3-Q results from the chemical properties of the quinones [26].

## Depurinating estrogen-DNA adducts: generators of cancer initiation

**Fig. 3:**
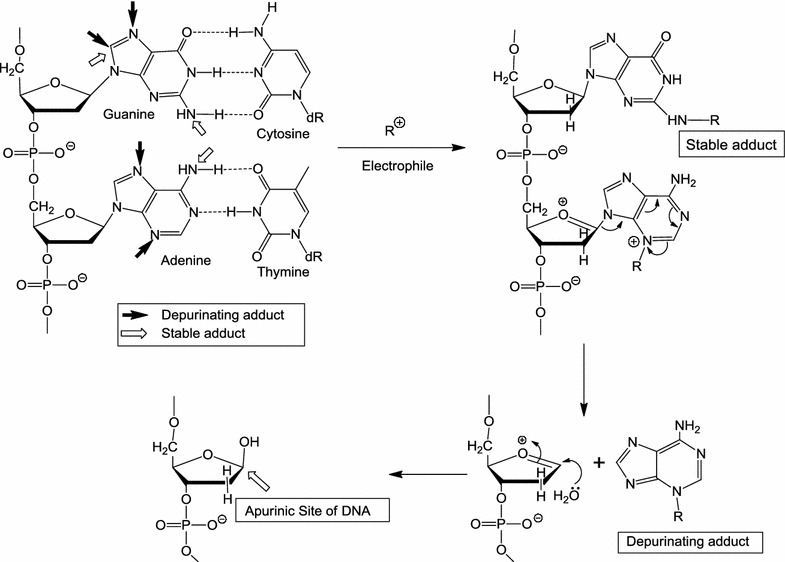
Formation of stable DNA adducts, and depurinating DNA adducts that generate apurinic sites

Carcinogens react with DNA to form two types of adducts: stable adducts and depurinating adducts. In the Watson–Crick DNA model (Fig. 3), the backbone is constituted by deoxyribose and phosphate, the Gua is hydrogen-bonded to cytosine, and Ade is hydrogen-bonded to thymine. The Gua has one exocyclic NH_2_ group that can react with electrophiles to form a stable adduct (Fig. 3, hollow arrow). If reaction occurs at the N-7 and sometimes C-8 of Gua, depurinating adducts are formed (Fig. 3, solid arrows). In the case of Ade, reaction of an electrophile at the exocyclic NH_2_ group forms a stable adduct (Fig. 3, hollow arrow), whereas depurinating adducts are obtained after reaction at the N-3 and N-7 sites (Fig. 3, solid arrows). Following reaction at the N-3 of Ade, destabilization of the glycosyl bond occurs via formation of an intermediate oxocarbenium ion with subsequent depurination and generation of an apurinic site in the DNA [54].

Cancer researchers have focused on stable adducts, which remain in DNA unless removed by repair. These adducts are routinely detected and quantified by the ^32^P-postlabeling technique, but their structure has not always been identified.

Stable adducts are formed when electrophilic carcinogenic compounds react with the exocyclic amino group of Ade or Gua [25]. If formation of adducts occurs at the N-3 or N-7 of Ade, or the N-7 of Gua, the most nucleophilic sites in Ade and Gua [55], destabilization of the glycosyl bond and subsequent depurination of the adduct from DNA takes place [20, 22, 25]. The critical relevance of these depurinating adducts is still not recognized by researchers 20 years after their discovery [56].

Evidence that depurinating DNA adducts play the predominant role in cancer initiation was first obtained from a correlation between the levels of depurinating polycyclic aromatic hydrocarbon-DNA adducts and oncogenic Harvey (H)-*ras* mutations in mouse skin papillomas [56, 57]. The very potent carcinogens 7,12-dimethylbenz[*a*]anthracene [58] and dibenzo[*a,l*]pyrene [59, 60] form predominantly depurinating Ade adducts and induce an A to T transversion in codon 61 of the H-*ras* oncogene. Instead, benzo[*a*]pyrene yields approximately twice as many Gua depurinating adducts as Ade depurinating adducts in mouse skin [61], and twice as many codon 13 G to T transversions as codon 61 A to T transversions [56, 61, 62].

A similar correlation between the sites of formation of depurinating DNA adducts and H-*ras* mutations was observed in mouse skin and rat mammary glands treated with E_2_-3,4-Q [63, 64].

### E_1_(E_2_)-3,4-quinones and E_1_(E_2_)-2,3-quinones

**Fig. 4:**
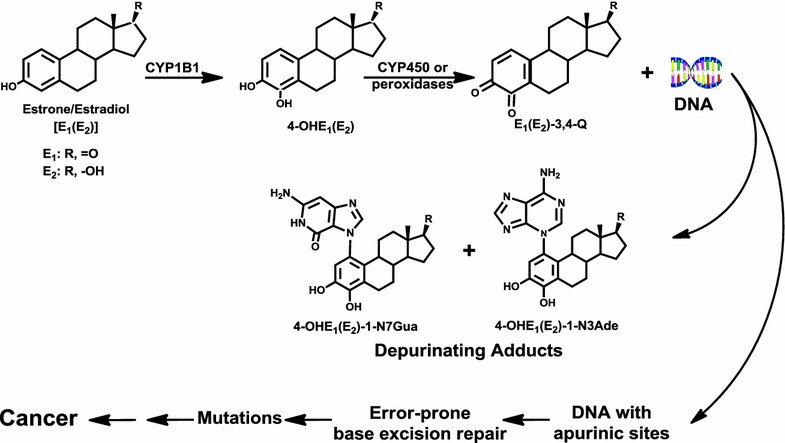
Major metabolic pathway in cancer initiation by estrogens

The predominant cancer initiating pathway (97 %) derives from E_1_(E_2_)-3,4-Q and is shown in Fig. 4 [26]. E_1_ and E_2_ are metabolically converted to 4-OHE_1_(E_2_) by CYP1B1. Oxidation of the catechol estrogens leads to the corresponding E_1_(E_2_)-3,4-Q, which can react with DNA to form small amounts of stable adducts (1 %) remaining in the DNA and preponderant amounts of the depurinating adducts 4-OHE_1_(E_2_)-1-N3Ade and 4-OHE_1_(E_2_)-1-N7Gua (97 %), which detach from DNA leaving behind DNA with apurinic sites [26]. Possible errors in the repair of these sites can lead to the critical mutations initiating many common human cancers [63, 64].

**Fig. 5:**
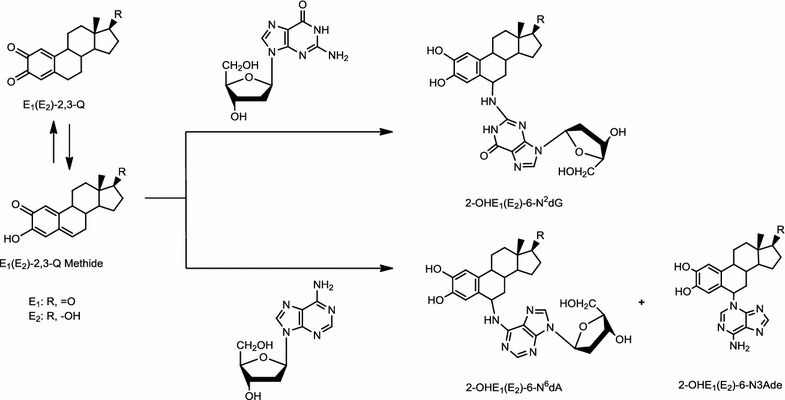
Reaction of E_1_(E_2_)-2,3-Q with dG or dA to form the stable 2-OHE_1_(E_2_)-6-N^2^dG or 2-OHE_1_(E_2_)-6-N^6^dA adducts (minor), respectively, and the depurinating 2-OHE_1_(E_2_)-6-N3Ade adducts (major)

E_1_(E_2_)-2,3-Q form a much lower amount (2 %) of the depurinating adducts 2-OHE_1_(E_2_)-6-N3Ade by 1,6-Michael addition (Fig. 5) [26]. This product is obtained after tautomerization of the E_1_(E_2_)-2,3-Q to E_1_(E_2_)-2,3-Q methide [65]. The E_1_(E_2_)-2,3-Q form 10 to 50 times higher levels of stable DNA adducts than E_1_(E_2_)-3,4-Q [20, 24]. The level of stable adducts formed by E_1_(E_2_)-2,3-Q is still lower than the level of the depurinating adducts 2-OHE_1_(E_2_)-6-N3Ade [21, 26].

**Fig. 6:**
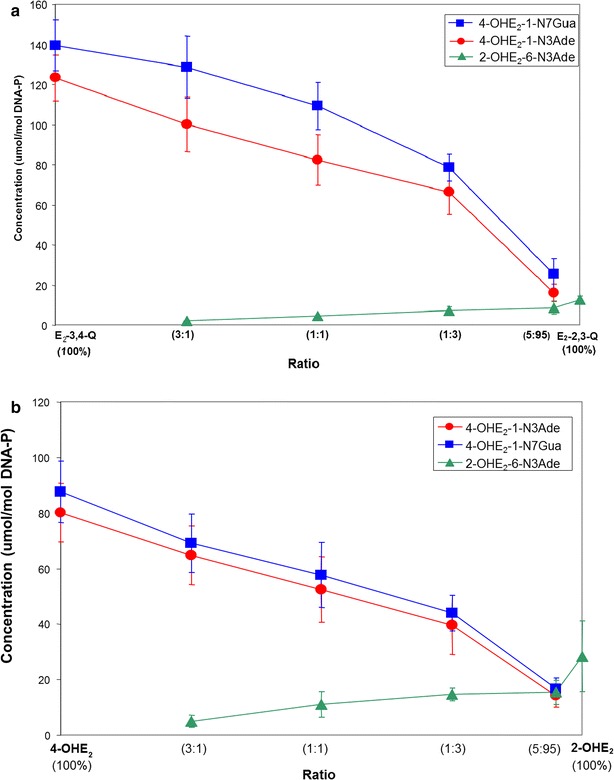
Depurinating adducts formed by mixtures of **a** E_2_-3,4-Q and E_2_-2,3-Q at different ratios after 10 h of reaction with DNA. The level of stable adducts formed in the mixtures ranged from 0.1 to 1 % of total adducts; and **b** 4-OHE_2_ and 2-OHE_2_ in the presence of tyrosinase at different ratios after 10 h of reaction with DNA. The level of stable adducts formed in the mixtures ranged from 0.1 to 0.7 % of total adducts [26]

The effectiveness of the E_1_(E_2_)-3,4-Q versus E_1_(E_2_)-2,3-Q to form depurinating adducts has been determined by reacting a mixture of E_2_-3,4-Q and E_2_-2,3-Q with DNA at different ratios. To achieve comparable levels of depurinating adducts, the mixture needs to contain 95 % E_2_-2,3-Q and 5 % E_2_-3,4-Q (Fig. 6a) [26].

Similar results are obtained from mixtures of 4-OHE_2_ and 2-OHE_2_ oxidized by tyrosinase in the presence of DNA (Fig. 6b). These results demonstrate the effectiveness of E_2_-3,4-Q to react with DNA in the formation of depurinating adducts compared to E_2_-2,3-Q.

The levels of depurinating DNA adducts formed by the catechol estrogen quinones [26] are in agreement with the greater carcinogenic activity of 4-OHE_1_(E_2_) compared with the borderline carcinogenic activity of 2-OHE_1_(E_2_) [66–68].

## Imbalance of estrogen metabolism in cancer initiation

The metabolism of estrogens through the catechol estrogen pathway is characterized by homeostasis, a balanced set of activating and protective enzymes. Homeostasis minimizes the metabolic oxidation of catechol estrogens to catechol quinones and their reaction with DNA (Fig. 2). Disruption of homeostasis in the metabolism of estrogens, with excessive production of estrogen quinones and depurinating estrogen-DNA adducts, can lead to the initiation of cancer. A variety of endogenous and exogenous factors can disrupt estrogen homeostasis.

One factor that can imbalance estrogen metabolism is the excessive synthesis of estrogens by overexpression of CYP19 (aromatase) in target tissues (Fig. 2) [69–71]. A second factor that can imbalance estrogen homeostasis is the presence of unregulated sulfatase that converts excessive stored E_1_-sulfate into E_1_ (Fig. 2) [72, 73]. A third factor in imbalance is the production of high levels of 4-OHE_1_(E_2_), due to overexpression of CYP1B1, which converts E_1_(E_2_) predominantly to 4-OHE_1_(E_2_) (Fig. 2) [45–47, 74, 75]. Higher levels of 4-OHE_1_(E_2_) can give rise to higher levels of the strongest ultimate carcinogenic metabolites, E_1_(E_2_)-3,4-Q. An analogous effect can be produced by a lack or low level of COMT activity due to polymorphic variation [49, 76]. Insufficient activity of this enzyme would be translated into low levels of methylation of 4-OHE_1_(E_2_) and subsequent increase in the competitive oxidation of 4-OHE_1_(E_2_) to E_1_(E_2_)-3,4-Q (Fig. 2). Higher levels of E_1_(E_2_)-3,4-Q can also be obtained by polymorphism in quinone reductase (NQO1) which leads to decreased conversion of quinones into catechols (Fig. 2) [77]. Furthermore, low cellular levels of GSH, which reacts efficiently with the quinones, can leave higher levels of E_1_(E_2_)-3,4-Q available.

Imbalances in estrogen metabolism have also been observed in animal models for estrogen carcinogenicity: the prostate of Nobel rats [78], the kidney of male Syrian golden hamsters [79] and the mammary gland of ER-α knockout mice [80]. Imbalance of estrogen homeostasis can also be seen by comparing analyses of breast tissue from women with and without breast cancer [81]. In non-tumor breast tissue from women with breast carcinoma, the levels of 4-OHE_1_(E_2_) were nearly four-times higher than the levels in breast tissue from women without breast cancer. Further evidence of imbalance in estrogen homeostasis derives from the greater expression of estrogen-protective enzymes, COMT and NQO1 (Fig. 2), in women without breast cancer and greater expression of estrogen-activating enzymes, CYP19 and CYP1B1 (Fig. 2), in breast tissue of women with breast cancer [82].

Imbalance in estrogen metabolism can also be triggered by environmental factors. These factors include substances we ingest by mouth, skin and nose. It is logical to hypothesize that these environmental compounds are capable of changing estrogen metabolism, leading to increased formation of catechol estrogen quinones. Dioxin, for example, induces expression of the activating enzyme CYP1B1 [74, 75] (Fig. 2). This compound is not carcinogenic by itself, but makes the estrogens become carcinogenic by disrupting their metabolic homeostasis.

## Depurinating estrogen-DNA adducts, the biomarkers of risk for women with and without breast cancer

Development of biomarkers for cancer risk has been a major goal in cancer research for decades. The ratio of the depurinating estrogen-DNA adducts 4-OHE_1_(E_2_)-1-N3Ade, 4-OHE_1_(E_2_)-1-N7Gua and 2-OHE_1_(E_2_)-6-N3Ade to their respective catechol estrogen metabolites and catechol estrogen conjugates provides a biomarker of risk that is related to the initiating step of breast and other prevalent types of human cancer.

$$ \begin{aligned}{\rm{ratio}} &= \left( \frac{{4{\text{-}}{\rm{OHE}}_{1} \left( {{\rm{E}}_{2} } \right){\text{-}}1{\text{-}}{\rm{N}}3{\text{Ade}} + 4{\text{-}}{\rm{OHE}}_{1} \left( {{\rm{E}}_{2} } \right){\text{-}}1{\text{-}}{\text{N}}7{\rm{Gua}}}}{{4{\text{-}}{\text{catechol estrogens}}\, + \,4{\text{-}}{\text{catechol estrogen}}\,{\text{conjugates}}}} \right. \\ &+  \left. \frac{{2{\text{-}}{\rm{OHE}}_{1} \left( {{\rm{E}}_{2} } \right){\text{-}}1{\text{-}}{\rm{N}}3{\rm{Ade}}}}{{2{\text{-}}{\text{catechol estrogens}}  +  2{\text{-}}{\text{catechol estrogen}}\,{\text{conjugates}}}} \right) \times 1000 \end{aligned}$$

**Fig. 7:**
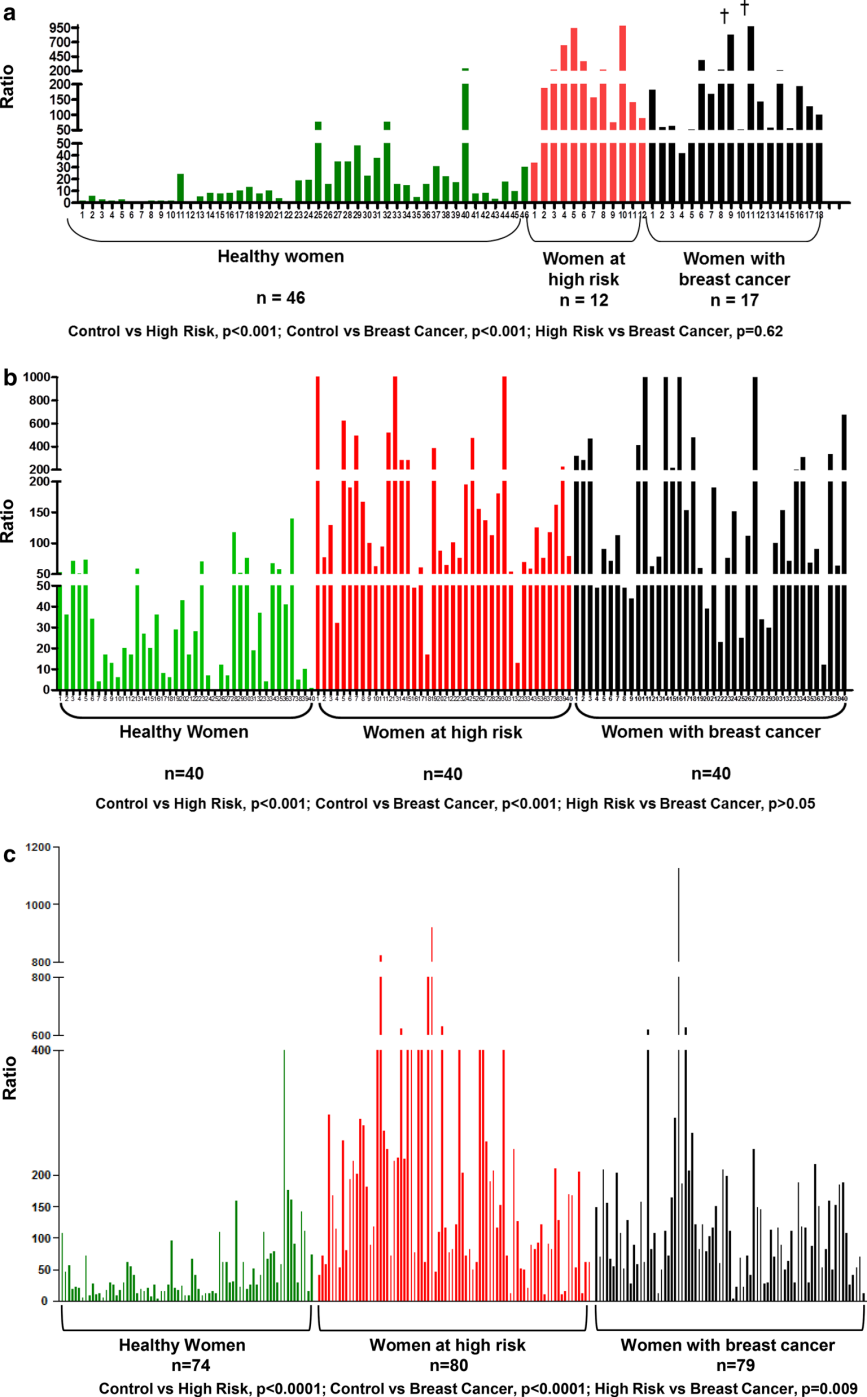
Ratios of depurinating estrogen-DNA adducts to catechol estrogen metabolites and catechol estrogen conjugates in **a** first study [83]: urine of healthy women, high risk women and women with breast cancer; **b** second study [84]: urine of healthy women, high risk women and women with breast cancer; **c** third study [85]: serum of healthy women, high risk women and women with breast cancer

Three case–control studies have been conducted in women diagnosed with breast cancer, as well as women at high risk or normal risk for the disease (Fig. 7) [83–85]. The high-risk women were identified by using the Gail model score to estimate a 5-year risk greater than 1.66 % [86]. Calculation of the Gail model score is based on age, age at menarche, age at first birth, prior breast biopsies or atypical hyperplasia, and number of first-degree relatives with breast cancer.

In the first two studies [83, 84], a spot urine sample (~50 ml) was collected from each subject. An aliquot of the sample was partially purified by solid-phase extraction and analyzed for 38 catechol estrogen metabolites, catechol estrogen conjugates and depurinating estrogen-DNA adducts. The estrogen analytes were identified and quantified by using ultraperformance liquid chromatography/tandem mass spectrometry, and the ratio (see equation above) was calculated for each subject (Fig. 7a, b). In the first study of 46 normal-risk women, 12 high-risk women and 17 women diagnosed with breast cancer, the ratios in the high risk (p < 0.001) and breast cancer (p < 0.001) were significantly higher than the ratios in the normal-risk women (Fig. 7a) [83]. Similar differences were observed in the second study between 40 normal-risk women, 40 high-risk women and 40 women with breast cancer (both p < 0.001) (Fig. 7b) [84].

In the third study, serum was collected from each of the 74 normal-risk women, 80 high-risk women and 79 women diagnosed with breast cancer (Fig. 7c) [85]. Once again, the ratio of adducts to metabolites and conjugates was significantly lower in the women at normal risk, compared to the high-risk and breast cancer women (both p < 0.001).

In all three studies, the high ratios arose from high levels of adducts and low levels of metabolites and conjugates, although in some samples the levels of adducts were average, but the levels of metabolites and conjugates were very low [83–85], yielding a similar ratio in both cases. Overall, the high ratio of depurinating estrogen-DNA adducts to the catechol estrogen metabolites and catechol estrogen conjugates is a biomarker of risk for breast cancer.

Since estrogens initiate breast cancer by a genotoxic mechanism, the observation of higher levels of estrogen-DNA adducts in women at high risk for breast cancer suggests that formation of these adducts is a causative factor in the etiology of breast cancer and not a consequence of the cancer itself.

Similar case–control studies were conducted with women diagnosed with ovarian cancer and healthy women [87], and women with thyroid cancer and healthy women [88]. In both cases, the women diagnosed with the disease had much higher ratios of depurinating estrogen-DNA adducts to catechol estrogen metabolites and conjugates. Similar results were obtained in case–control studies of men with prostate cancer [89] or with non-Hodgkin lymphoma [90].

We think that other prevalent types of cancer, which have not yet been investigated for depurinating estrogen-DNA adduct formation, are also initiated by estrogens. These cancers include brain, colon, endometrium, kidney, leukemia, lung of non-smokers, melanoma, myeloma, pancreas and testis.

## Prevention of cancer initiation by *N*-acetylcysteine and resveratrol acting as antioxidants, enzyme modulators and inhibitors of depurinating estrogen-DNA adduct formation

**Fig. 8:**
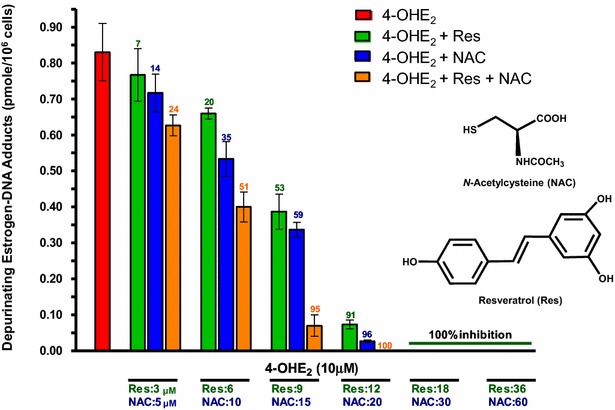
Ability of NAC, Res or their combination to block formation of depurinating estrogen-DNA adducts in MCF-10F human breast epithelial cells treated with 4-OHE_2_. The numbers on *bars* are the percentage of the inhibition of the depurinating estrogen-DNA adducts compared to treatment with 4-OHE_2_ alone [106]

The metabolism of estrogens in the catechol estrogen pathway is regulated by homeostasis, a balanced set of activating and protective enzymes. Homeostasis can be maintained or re-established by the use of specific compounds, *N*-acetylcysteine (NAC) and resveratrol (Res), which are particularly effective in blocking formation of estrogen-DNA adducts [91]. NAC is the acetyl derivative of the amino acid cysteine (Fig. 8), which is one component of the tripeptide GSH. Res, which is the 3,5,4′-hydroxy stilbene (Fig. 8), is a natural antioxidant present in grapes, wine, peanuts and other plants. NAC and Res can prevent oxidative and/or electrophilic damage to DNA by inhibiting formation of the electrophilic catechol estrogen quinones and/or reacting with them.

The anticarcinogenic properties of NAC are attributed to multiple protective mechanisms, such as its nucleophilicity, antioxidant activity and inhibition of DNA adduct formation [92, 93]. Hydrolysis of NAC by acylase in the liver and gut yields cysteine, one of the precursors in the synthesis of intracellular GSH. The presence of cysteine guarantees replenishment of this crucial tripeptide. Changes in GSH homeostasis have been implicated in the etiology and progression of cancer and other human diseases [94]. GSH cannot be used as a preventive agent because it does not cross cell membranes. The use of cysteine as a preventive agent is limited by its toxicity. NAC, instead, has very low toxicity and it can cross the blood–brain barrier [92, 93]. NAC reacts efficiently with the electrophilic E_1_(E_2_)-3,4-Q [95, 96] to prevent their reaction with DNA to form adducts (Fig. 2). Furthermore, NAC reduces catechol estrogen semiquinones to catechol estrogens (Fig. 2) [97] and prevents malignant transformation of the human MCF-10F cells [98], as well as the mouse E6 mammary cells treated with 4-OHE_2_ [99].

Res exerts chemopreventive effects in various in vitro and in vivo systems [100, 101]. These properties are attributed to the easy hydrogen abstraction from the 4′-OH bond with formation of an oxy radical [102]. The easy abstraction is due to the great resonance stabilization energy of the oxy radical intermediate. Res is a modulator of CYP1B1 [74, 75, 103] and an inducer of quinone reductase (Fig. 2) [75, 104]. Res also reduces estrogen semiquinones to catechol estrogens (Fig. 2) [75]. When MCF-10F cells are cultured in the presence of 4-OHE_2_ and Res, formation of depurinating estrogen-DNA adducts is inhibited in a dose-dependent manner [75, 105]. To investigate whether the inhibitory effects of NAC and Res on the formation of estrogen-DNA adducts are additive or synergistic, MCF-10F cells were cultured in the presence of 4-OHE_2_ plus NAC or Res or NAC and Res together (Fig. 8) [106]. It was found that the effects of NAC and Res combined were additive in inhibiting formation of the depurinating estrogen-DNA adducts (p < 0.0001) [106]. NAC and Res had similar inhibitory effects at low concentrations, but the effects of Res were about 50 % greater than those of NAC at high concentrations.

**Fig. 9:**
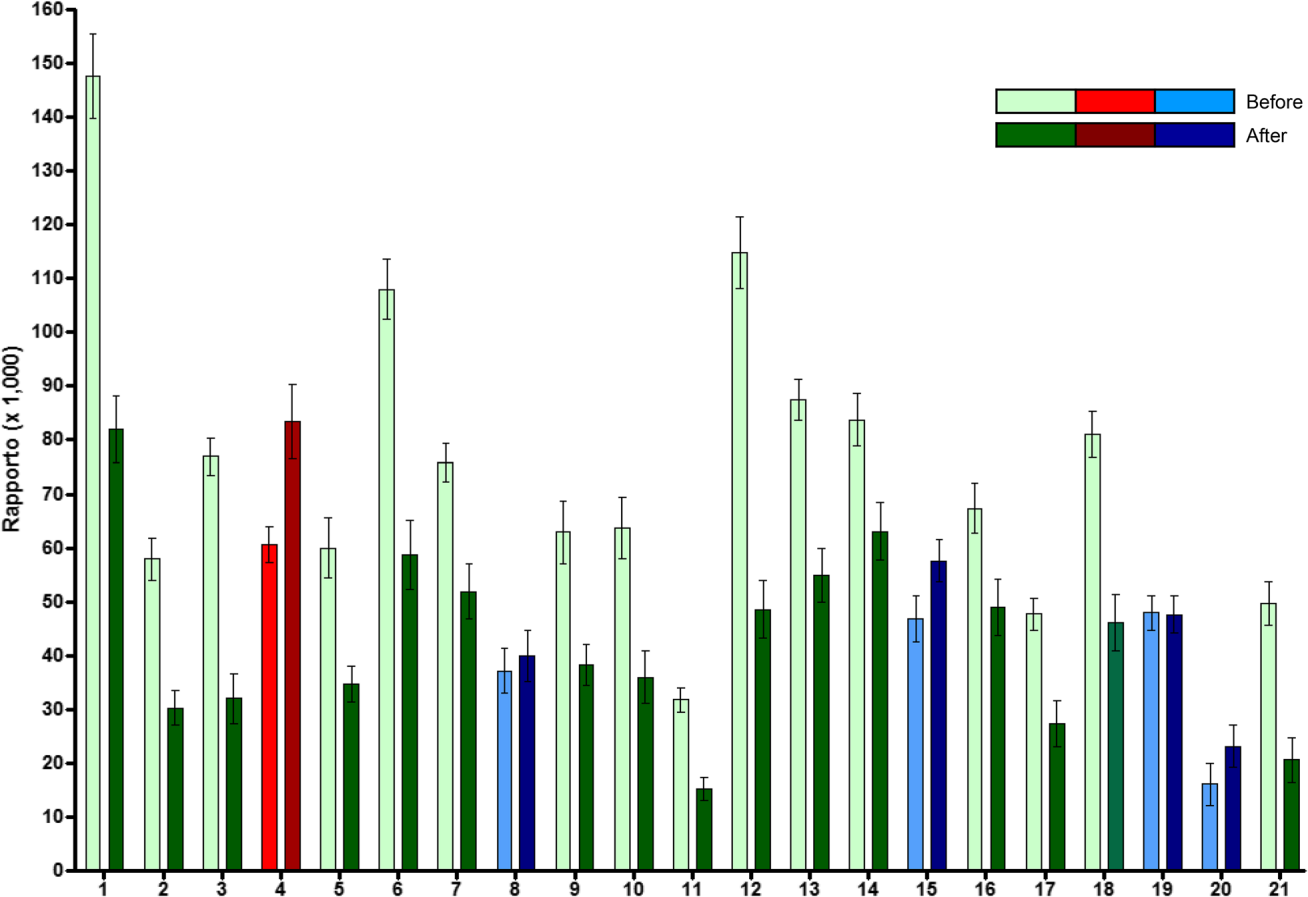
Assessment of depurinating estrogen-DNA adduct ratios before women began treatment with the Healthy Breast Protocol and after having been on the treatment for 3 months. *Green bars* represent women whose adduct ratios decreased, *blue bars* represent women whose adduct ratios remained the same and the *red bars* represent a woman whose adduct ratio increased over the course of the study [107]

A Healthy Breast Protocol that included NAC and Res was administered to women [107]. A group of 21 women (ages 30–70), who had never been diagnosed with cancer, participated in a study of the Healthy Breast Protocol [107]. They followed the treatment daily for 3 months and provided a spot urine sample before starting the treatment and after the 3 month period. The urine samples were analyzed for catechol estrogen metabolites and conjugates, and depurinating estrogen-DNA adducts by ultraperformance liquid chromatography/tandem mass spectrometry, and the ratio of adducts to metabolites and conjugates was calculated for each sample (Fig. 9). Of the 21 women participants, 16 experienced a decrease (green bars) in the ratio of adducts to metabolites and conjugates, four remained the same (blue bars) and one had an increase (red bars). The decrease in the ratio after treatment was statistically significant (p < 0.03) [107]. These results indicate that a treatment including NAC and Res can reduce depurinating estrogen-DNA adduct levels in people. This preventive approach does not require knowledge of the genes involved or the complex series of events that follow cancer initiation.

In summary, NAC and Res are both able to reduce estrogen semiquinones to catechol estrogens [75, 97]. Furthermore, NAC keeps the cell replenished with GSH and reacts efficiently with the potential carcinogens, catechol estrogen quinones (Fig. 2). Res induces the enzyme quinone reductase and modulates the CYP1B1 activity (Fig. 2). Thus, NAC and Res, by inhibiting formation of depurinating estrogen-DNA adducts, maintain homeostasis in the metabolism of estrogens.

## Conclusions

Metabolism of estrogens via the catechol estrogen pathway is characterized by homeostasis, a balanced set of activating and protective enzymes (Fig. 2). Under these conditions, formation of the catechol estrogen quinones, the ultimate carcinogenic metabolites of estrogens, is minimized. These compounds are not available to react with DNA; therefore, cancer cannot be initiated. When homeostasis is disrupted, however, excessive oxidation of catechol estrogens to quinones occurs. The quinones can react with DNA to form predominantly the depurinating adducts 4-OHE_1_(E_2_)-1-N3Ade and 4-OHE_1_(E_2_)-1-N7Gua. The apurinic sites derived from the loss of these adducts from DNA lead to the mutations that can initiate cancer.

Knowledge of the mechanism of cancer initiation by estrogens suggests that prevention of cancer can be achieved by blocking formation of the depurinating estrogen-DNA adducts. If the initiation of cancer is blocked, promotion, progression and development of the disease would be prevented. A variety of evidence suggests that cancer prevention could be achieved by use of the dietary supplements NAC and Res. Thus, use of these two dietary supplements could prove to be a widely applicable approach to cancer prevention.

## Abbreviations

CAT: catechol
COMT: catechol-*O*-methyltransferase
CYP: cytochrome P450
CYP19: aromatase
DES: diethylstilbestrol
E_1_: estrone
E_2_: estradiol
ER: estrogen receptor
GSH: glutathione
H: Harvey
HES: hexestrol
NAC: *N*-acetylcysteine
NQO1: quinone reductase
Res: resveratrol
